# Supplementary material for: Direct medical cost of adult Covid-19 ınpatients and ıts determinants at a university hospital
Source: PLoS One. 2025 Apr 7;20(4):e0319762. doi: 10.1371/journal.pone.0319762 (PMC11975136; doi:10.1371/journal.pone.0319762)
Supplement: Attachment — Submitted filename: response to reviewers.docx [file pone.0319762.s003.docx]

**Response to reviewers**

First of all, we thank reviewers’ attention and suggestions about our manuscript. All of them has been carefully considered. The revisions we have made are explained below and are visable on revised manuscript with track changes.

1. **References**
a. References in the reference list have been updated to be accessible.
2. **Methodological Clarity and Comparisons**
a. Explanation of why the study period or specific variables were chosen has been added.
b. The methods section has been expanded to include detailed descriptions of costing criteria and approaches used in cited studies.
c. Significant differences in methodologies and their impact on results has been explained.
3. **Contextual Differences**
a. Concrete examples are included to illustrate how differences in health policies, resource allocation, and treatment protocols affect costs.
4. **Statistical Details and Consistency in Data Presentation**
a. Posthoc analyses were performed to understand which groups accounted for the observed differences (table 3).
b. Throughout the article, justification for using different cost metrics was provided.
c. The conformity of numerical formatting to international standards was reviewed.
5. **In-depth Discussion of Sources**
a. The discussion has been expanded on resource allocation strategies in these countries and how they differ from Turkey.
b. The strengths and weaknesses of the cited studies, sample sizes, and how the scope of each study might affect their findings were examined.
6. **Positioning of Study Findings**
a. The unique contributions of the study has been clearly stated.
b. Emphasis how the findings from Turkey compare to global trends and what these comparisons reveal about health care costs in different contexts.
7. **Structure and Flow**
a. Text has been revised to eliminate repetitions and increase consistency
8. **Additional Suggestions for Improvement**

**Terminological Clarity:**

a. Key terms has been clearly defined in the introduction or methods section.

**Comprehensive Conclusion:**

a. Conclusion has been revised to synthesize the key findings of the study with insights from global comparisons

**Point to point revisions suggested by reviewer 2**

a. Instead of "COVID-19" "Covid-19" was written.
b. The odds ratios (OR) presented in the summary are reported with confidence intervals.
c. Brief information has been added about the medical costs of Covid-19. References about the economic burden of Covid-19 on healthcare systems in some countries has been added.
d. A subheading for Inclusion Criteria has been added.
e. Which services were selected and why has been explained.
f. References in the reference list have been updated to be accessible.
g. The wide confidence intervals were discussed in the limitations section.
h. Explanation of why we chose to use the median value as the cut-off point for multivariate analysis has been added. (Since the dependent variable, the per capita cost of COVID-19, did not comply with normal distribution. Therefore, in the multivariate logistic regression analysis, the per capita cost of COVID-19 was categorized from the median value)
i. The last paragraphs under the heading Strengths and Limitations have been moved to the conclusion.
